# *ERBB4* exonic deletions on chromosome 2q34 in patients with intellectual disability or epilepsy

**DOI:** 10.1038/s41431-021-00815-y

**Published:** 2021-02-18

**Authors:** Zerin Hyder, Wim Van Paesschen, Ataf Sabir, Francis H. Sansbury, Katherine B. Burke, Naz Khan, Kate E. Chandler, Nicola S. Cooper, Ronnie Wright, Edward McHale, Hilde Van Esch, Siddharth Banka

**Affiliations:** 1 Manchester Centre for Genomic Medicine, St Mary’s Hospital, Manchester University NHS Foundation Trust, Manchester, UK; 2 Department of Neurology, University Hospitals Leuven, Leuven, Belgium; 3 Laboratory for Epilepsy Research, Katholieke Universiteit Leuven, Leuven, Belgium; 4 West Midlands Regional Genetics Service, Birmingham Women’s and Children’s NHS Foundation Trust, Birmingham, UK; 5 All Wales Medical Genomics Service, NHS Wales Cardiff and Vale University Health Board, Institute of Medical Genetics, University Hospital of Wales, Cardiff, UK; 6 Division of Evolution & Genomic Sciences, School of Biological Sciences, Faculty of Biology, Medicine and Health, University of Manchester, Manchester, UK; 7 Center for Human Genetics, University Hospitals Leuven, University of Leuven, Leuven, Belgium

**Keywords:** Chromosome abnormality, Genetic testing, Neurodevelopmental disorders

## Abstract

*ERBB4* encodes the tyrosine kinase receptor HER4, a critical regulator of normal cell function and neurodevelopmental processes in the brain. One of the key ligands of HER4 is neureglin-1 (NRG1), and the HER4-NRG1 signalling pathway is essential in neural crest cell migration, and neuronal differentiation. Pharmacological inactivation of HER4 has been shown to hasten the progression of epileptogenesis in rodent models, and heterozygous *ERBB4* null mice are shown to have cognitive deficits and delayed motor development. Thus far there is only a single case report in the literature of a heterozygous *ERBB4* deletion in a patient with intellectual disability (ID). We identified nine subjects from five unrelated families with chromosome 2q34 deletions, resulting in heterozygous intragenic loss of multiple exons of *ERBB4*, associated with either non-syndromic ID or generalised epilepsy. In one family, the deletion segregated with ID in five affected relatives. Overall, this case series further supports that haploinsufficiency of *ERBB4* leads to non-syndromic intellectual disability or epilepsy.

## Introduction

Human *ERBB4* is located on chromosome 2q34, has 28 exons, encodes at least five protein coding transcripts and is highly constrained against loss-of-function variants, with a pLI (probability of loss-of-function intolerance) score of 1 [1]. It encodes a tyrosine kinase receptor HER4, a crucial regulator of cell function and neurodevelopmental processes in the brain. A vital ligand of HER4 is neureglin-1 (NRG1), and the HER4-NRG1 signalling pathway is critical in the migration of neural crest cells, and neuronal and glial differentiation [2]. HER4 is expressed in many adult and foetal tissues, with high expression in the brain and heart [3, 4].

Kasnauskiene et al. described a 15-year-old male patient with hyperactivity, intellectual disability (ID) and severe speech delay with a de novo 958 kb 2q34 deletion resulting in loss of exons 1–3 of *ERBB4* (OMIM 600543) [5]. Backx et al. reported a de novo balanced translocation t(2;6)(q34;p25.3) in a girl with early onset myoclonic encephalopathy and profound psychomotor delay [6]. The 2q34 breakpoint was located between exons 1 and 2 of *ERBB4*, which was proposed to be the major driver of the phenotype. Walsh et al. described a 399 kb deletion of the distal region of *ERBB4* in a patient with schizophrenia, resulting in loss of exons 20–28 [7]. Array comparative genomic hybridisation (array-CGH) in a large cohort of patients with autism spectrum disorder identified two patients with intronic deletions of *ERBB4* [8]. A meta-analysis by Feng et al. identified an *ERBB4* intronic single nucleotide polymorphism, rs707284, in association with schizophrenia [odds ratio = 0.91, 95% CI: 0.83–0.99, *P*  = 0.035] [9]. Takahashi et al. identified heterozygous *ERBB4* missense variants in three separate families with amyotrophic lateral sclerosis (ALS) [10]. Although *ERBB4* variants have been implicated in several neurological phenotypes, their significance has not been proven conclusively.

## Methods

### Case ascertainment

Informed consent was obtained from the probands or guardians of all investigated subjects. Family 1 was ascertained at the Manchester Centre for Genomic Medicine. Written consent was from obtained from Family 1 (Research Ethics Committee (REC) number 11/H1003/3). Additional cases were ascertained through the DECIPHER (Database of Chromosomal Imbalance and Phenotype in Humans using Ensembl Resources) (accessed 9th February 2020) database, by interrogating for high-confidence deletions exclusively affecting *ERBB4* in individuals with no other pathogenic or potentially pathogenic Copy number variants (CNVs) [11]. Clinical phenotypes were collected using a bespoke proforma. The logarithm of odds (LOD) score was calculated in Family 1 according to the formula LOD = log10$\frac{{{\it{\Theta}} ^{\mathrm{R}}(1 \,-\, {\it{\Theta}} ^{{\mathrm{NR}}})}}{{({\it{\Theta}} \,=\, 0.5)^{{\mathrm{R}} \,+\, {\mathrm{NR}}}}}$.

### Microarray and fluorescent in situ hybridisation

High-resolution whole-genome array-CGH was performed using DNA extracted from peripheral blood using the Oxford Gene Technology Cytosure constitutional version 3 Array, to investigate Families 1, 2 and 5. For the assessment of Family 3, array-CGH was carried out using the BlueGnome 8x60k v2.0 ISCA platform. Test DNA was referenced against same-sex control DNA and data was analysed in BlueFuse Multi v4.1. The 180k Cytosure ISCA v2 array was used to assess the proband in Family 4. Where required, 2q34 fluorescence in situ hybridisation (FISH) studies were performed using standard techniques.

## Results

### Case report

#### Family 1

**Fig. 1:**
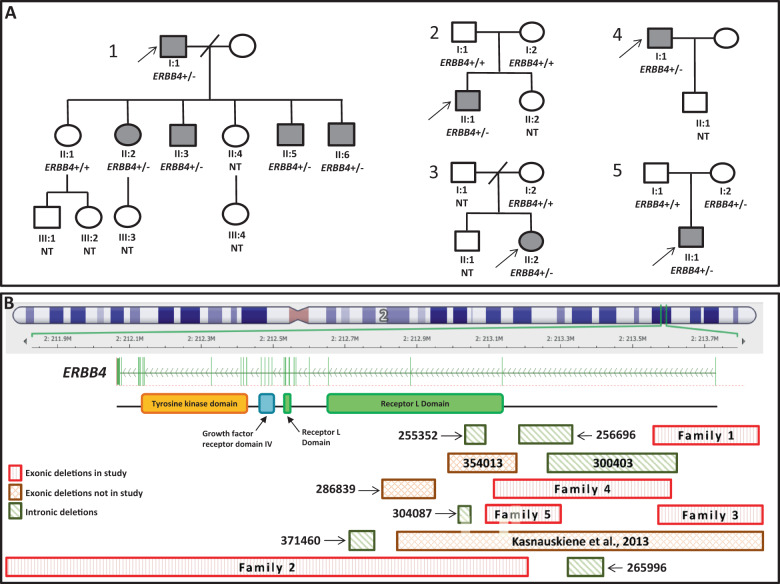
Pedigrees of families described in this paper and 2q34 deletions that exclusively involve *ERBB4*. **A** Pedigrees of Families 1–5. Shaded boxes depict subjects with intellectual disability or epilepsy; NT indicates not tested. *ERBB4+/+* indicates two wild-type alleles*; ERBB4+/*− indicates the presence of heterozygous ERBB4 exonic deletion. **B** Ideogram of chromosome 2 depicting DECIPHER deletions. Exonic deletions included in our study are illustrated with red boxes with vertical lines; exonic deletions not included in our study are orange boxes with hatched lines; and purely intronic deletions are green boxes with diagonal lines.

*Patient I:1* was a 61-year-old male referred to the clinical genetics service with ID. He demonstrated minor problems in attention, mild linguistic difficulties and problems with reading, writing, spelling and calculation. A detailed neuropsychological assessment identified primary problems with executive functioning, though his memory was relatively good and there were no notable problems with visuospatial functioning. He had six children, four of whom have ID (Fig. 1A). His second child (Family 1, *Patient II:2* in Fig. 1A), was born at 38 weeks gestation with a birth weight of 3.03 kg. There were no neonatal concerns. She was reported to have speech delay and mild motor delay. She sat by 7 months and walked by two and a half years. She was diagnosed with moderate to severe learning difficulties and attended a special educational needs school. At the age of 24 years, she could understand simple instructions and read short sentences. She required help with most activities of daily living including showering, toileting and dressing. Her behaviour was unpredictable with irritability and aggressive outbursts. She has one daughter, III:3, who had normal developmental milestones at the age of 2 years. *Patient II:3* was born at full term by normal delivery, with a birth weight of 2.72 kg. He had moderate speech delay. He sat by 7 months and walked by 18 months. He attended a special school. At the age of 23, he was noted to have unpredictable behaviour with episodes of aggression. *Patient II:5* is the fourth child of Patient 1-I. He spoke his first words by 3 years of age and walked by 18 months. He attended a special educational needs school. *Patient II:6* was born at full term with a birth weight of 3.17 kg. The pregnancy was complicated by maternal jaundice. He was able to speak in sentences by 7 years of age and walked by 2 years of age. He was noted to have severe behavioural problems and aggression. At the age of 16 years, he was studying Foundation level maths at College. All affected individuals including the proband were non-dysmorphic with normal growth parameters and a normal general and neurological examination. Individuals II:1 and II:4 in this family attained developmental milestones within normal limits, attended mainstream schools and did not have ID.

An array-CGH in Patient I:1 identified a chromosome 2q34 deletion of 276.5 kilobases (kb) encompassing exon 1 (numbered as in NM_005235.3 and demarcated from g.213403173_213403565 in GRCh 37) of *ERBB4* (NC_000002.11:g.(213170347_213255794)_(213532234_213581360)del) (DECIPHER ID: 271941). Further array-CGH and fluorescent in situ hybridisation (FISH) studies showed that the deletion was present in all affected individuals and absent in all unaffected individuals who were tested (Fig. 1A). The LOD score for the association of the deletion with the phenotype in this family was calculated as 1.81 (recombination factor (**ϴ**) = 0; the number of informative meiosis/individuals (*N*) = 6, and the number of recombinants (*R*) = 0).

### *ERBB4* exonic deletions identified from DECIPHER

We identified 12 additional deletions exclusively encompassing *ERBB4* from DECIPHER (Table 1) (Fig. 1). Of these, six deletions involved at least one exon of *ERBB4* and six were exclusively intronic. Out of six exonic deletions, two were shown to have occurred de novo, and one was maternally inherited. We collected more phenotype details in four of these families with exonic deletions (Families 2–5), which are summarised below as additional case reports.

**Table 1:** Genetic and clinical summary of deletions affecting *ERBB4* on chromosome 2q34.

**Exonic deletions of families described in this paper**						
**Case number**	**DECIPHER Accession number**	**Variant**	**Size**	**Exons or introns deleted**^**a**^	**Inheritance**	**Phenotype**
1	Family 1: 271941	NC_000002.11:g.(213170347_213255794)_(213532234_213581360)del	276.5 kb	Exon 1	Segregates in the family with the phenotype	Moderate speech delay, moderate intellectual disability; problems with executive functioning; linguistic difficulties; aggressive outbursts
2	Family 2: 323625	NC_000002.11:g.(211787769_211901530)_(213054251_213106838)del	1.15 Mb	Exons 2–28	De novo	Moderate speech delay; mild to moderate intellectual disability; aggressive outbursts
3	Family 3: 356496	NC_000002.11:g.(213170326_213255865)_(213532447-213581503)del	276.58 kb	Exon 1	Unknown	Moderate speech delay; moderate intellectual disability; aggressive outbursts; hyperactivity
4	Family 4: 331635	NC_000002.11:g.(212812342_212973847)_(213286482-213403172)del)	312.64 kb	Exon 2	Unknown	Childhood-onset tonic clonic/absence seizures
5	Family 5: 379018	NC_000002.11:g.(212885516_212945237)_(213106838_213170347)del	161.66 kb	Exon 2	Inherited from normal parent	Global developmental delay; repetitive behaviour; poor eye contact; dental crowding; pes planus
**Exonic deletions with limited clinical details**						
6	354013	NC_000002.11:g.(212885486_213010215)del	125 kb	Exon 2	Unknown	Severe expressive language delay
7	286839	NC_000002.11:g.(212772926_ 212840266)del	67.34 kb	Exon 3	De novo	Hypotonia, central sleep apnoea
**Intronic deletions**						
8	371460	NC_000002.11:g.(212671703_212707593)del	35.89 kb	Intron 3	Unknown	No information
9	256696	NC_000002.11:g.(213010307_213107207)del	96.9 kb	Intron 1	Inherited from normal parent	Intellectual disability, speech delay
10	304087	NC_000002.11:g. (212893498_212914269)del	20.77 kb	Intron 2	Paternally inherited	No information
11	255352	NC_000002.11:g.(212907456_212964613)del	57.16 kb	Intron 2	Inherited from normal parent	No information
12	265996	NC_000002.11(213107178_213170355)del	63.18 kb	Intron 1	Inherited from normal parent	Pulmonary stenosis, facial abnormality
13	300403	213054092-213292156 NC_000002.11:g.(213054092_213292156)del	238 kb	Intron 1	Unknown	Polycystic kidney dysplasia, postaxial polydactyly

^a^Exons are numbered as in NM_005235.3. Exon 1 is demarcated from g.213403173_213403565. Exons 2–28 follow this numbering.

### Additional case reports

#### Family 2

*Patient II:1* (Fig. 1A) was the second child of healthy, non-consanguineous parents of normal intellect. Pregnancy was complicated by a urinary tract infection in the third trimester. He was born at 41 weeks gestation by normal delivery with a birth weight of 3.71 kg. He manifested speech delay and required speech and language therapy from the age of 3 years. He was able to speak in short sentences by 4 years of age. He attended a mainstream school with extra support; his school age was 1 year behind his chronological age. At 11 years of age, he could speak in short sentences and understand simple instructions. He had some behavioural difficulties such as irritability and angry outbursts. On examination, he had no significant dysmorphic features. His head circumference was 54.2 cm (25th centile), height 149 cm (50–75th centile) and weight 40 kg (50–75th centile). Array-CGH in Patient II:1 identified a chromosome 2q34 deletion of 1.15 Mb encompassing exons 2–28 of *ERBB4* (NC_000002.11:g.(211787769_211901530)_(213054251_213106838)del). FISH studies performed on blood samples from both parents for the *ERBB4* deletion indicated that this CNV arose de novo in Patient II-1.

#### Family 3

*Patient II-2* (Fig. 1A) was the second child of unrelated parents. Her father was noted to have a history of learning difficulties and dyslexia at school. The proband was born at 39 + 3 weeks gestation with a birth weight of 3.28 kgs, by normal vaginal delivery. A heart murmur was detected after delivery, but an echocardiogram at 3 weeks of age showed a normal heart structure and no further cardiology follow-up was arranged. She achieved head control by 6 months, sat by 8 months and walked by 10 months. Her first words were at 2 years of age and she could speak in sentences by 5 years of age. She attended a special educational needs school and her behaviour was noted to be challenging with frequent outbursts. She was prescribed methylphenidate for attention deficit hyperactivity disorder. Examination aged 8 years showed a non-dysmorphic child with a head circumference of 49.5 cm (0.4th centile), height 130.5 cm (75th centile) and weight 25.7 kg (50th centile). Array-CGH detected a 276.58 kb deletion encompassing exon 1 of *ERBB4* (NC_000002.11:g.(213170326_213255865)_ (213532447-213581503)del) which was not present in her mother. Her father was not tested.

#### Family 4

*Patient I:1* (Fig. 1A) presented at the age of 14 years with generalised seizures. Following treatment with carbamazepine for 2 years, he subsequently remained seizure-free for 20 years. He later presented at the age of 36 with tonic clonic and absence seizures. His electroencephalogram showed generalised epileptic discharges, and his MRI brain scan showed no significant abnormalities. He was prescribed levetiracetam and lamotrigine and had not had further seizures on medication. There was no history of developmental delay or ID. His clinical examination and growth parameters were normal. He has one son who had a history of febrile convulsions. A 313 kb deletion was identified for Patient I:1 at chromosome 2q34 by array-CGH encompassing exon 2 of *ERBB4* (NC_000002.11:g.(212812342_212973847)_(213286482-213403172)del). No other family members were tested for this CNV.

#### Family 5

*Patient I:1* (Fig. 1A) was the first child of unrelated parents of normal intellect. Following an uncomplicated pregnancy, he was born at 41 + 6 weeks gestation by vaginal delivery with a birth weight of 3.29 kg. His gross motor milestones were normal, and he walked by 12 months of age. He had two words by the age of 2 years and eight words by the age of 3 years. He was noted to have poor eye contact and repetitive behaviour. At the age of 3 years and 10 months, he could join two words together, wave goodbye, had a palmar grip, and communicated using PECS (Picture Exchange Communication System) and Makaton. He had mild tooth crowding and pes planus. His head circumference was 51.2 cm (26th centile), height 101.8 cm (53rd centile) and weight 17.7 kg (76th centile). Array-CGH identified a chromosome 2q34 deletion of 162 kb encompassing exon 2 of *ERBB4* (NC_000002.11:g.(212885516_212945237)_(213106838_213170347)del). Testing for the Fragile X syndrome (OMIM 300624) did not identify *FMR1* expansion. The deletion was found to be inherited from unaffected mother.

### Intronic *ERBB4* deletions

From the six deletions from DECIPHER that were restricted to intronic regions, five cases were shown to be inherited, and in at least three instances the carrier parent was reported to be phenotypically normal. The deletion was not proven to have occurred de novo in any of the cases. The phenotype of the patients with intronic *ERBB4* deletions included developmental delay, ID, pulmonary stenosis, facial abnormality, polycystic kidney dysplasia and postaxial polydactyly.

## Discussion

Thus far, only one patient with ID and behavioural deficit with an intragenic *ERBB4* deletion has been reported [5]. Myoclonic encephalopathy, together with psychomotor delay, has been described in another subject with a translocation resulting in disruption of *ERBB4* [6]. Here, we present the largest case series of individuals with chromosome 2q34 deletions that exclusively affect *ERBB4*. Several observations point towards pathogenicity of the deletions involving exons of *ERBB4* in autosomal dominant ID or epilepsy. Firstly, although all individuals were ascertained on the basis of their genotype, all had developmental delay or epilepsy (Table 1). Secondly, the deletion in Family 1 segregated with the phenotype and in Family 2, the deletion was shown to have arisen de novo (Fig. 1A). *ERBB4* is integral to developmental processes, demonstrated by HER4 knockout mice that die mid-embryogenesis due to cardiac malformation [12]. Notably, heterozygous null mice show delayed motor development, memory deficits and altered cue use in Morris maze learning [13]. Recovery knockout rodent models that lack HER4 brain expression have a reduced number of interneurons in the brain and cortex that is not rescued after restoring HER4 expression in adults. These rodents manifest behavioural deficits such as hyperactivity, impaired social interaction and contextual fear memory [14]. The study of *ERBB4* function in rodent models suggests that almost all cells expressing HER4 modify the effects of gamma-Aminobutyric acid (GABA) in the cortex, hippocampus and basal ganglia [15]. HER4 clusters and associates with GABA receptors leading to receptor internalisation, indicating its role in regulation of the interneuron network and migration of GABA neurons, the dysfunction of which may lead to a reduced number or function of cortical GABAergic neurons [10, 16]. Tan et al. demonstrated acceleration in the progression of kindling-induced epileptogenesis in mouse models after HER4 inactivation or *ERBB4* deletion [17].

In addition to ID and epilepsy, the patients in our series demonstrated clinical features such as executive functioning difficulties, attention problems, linguistic difficulties, moderate speech delay, mild motor delay, irritability, aggressive outbursts, hyperactivity, and repetitive behaviour. We note that the affected individuals from Families 1 and 3 with similar deletions encompassing exon 1 of *ERBB4* share moderate speech delay, moderate ID and behavioural difficulties in common. Families 4 and 5 share deletions that include the Receptor L domain, with distinctive clinical features. Accordingly; there does not appear to be a clear correlation between the severity of ID/epilepsy or the presence of additional clinical features, and the size or location of deletion. The reason why some exonic deletions result in ID whereas others may cause epilepsy is not clear. Aetiological overlap between ID and epilepsy is well known and could potential explain the phenotypic differences across patients, as evidenced by dominant pathogenic variants in *HECW2* and *GRIN2B* [18, 19]. Intriguingly, the deletion of exons 20–28 in a patient with schizophrenia reported by Walsh et al. was shown to result in an alternative transcript deficient of the intracellular kinase domain [7]. They proposed that the alternative transcript could cause a dominant negative effect and thus disrupt neuronal migration and synaptic neurotransmission. In vitro functional studies of ALS-causing mutant *ERBB4* have been shown to result in reduced autophosphorylation of HER4 after stimulation with NRG1 [10]. Patient II:1 of Family 2 exhibited a large deletion of exons 2-28 encompassing both the Receptor L (ligand-binding) domains and tyrosine kinase domain of *ERBB4;* with a distinct phenotype to that reported by Walsh et al. Although haploinsufficiency appears to be the most likely basis of the cases presented here, we cannot exclude other mechanisms without functional studies.

Interestingly, the *ERBB4* deletion in Family 5 was found to be maternally inherited, though maternal intellect in I:2 was reportedly normal. It is possible that reduced penetrance and variable expressivity associated with *ERBB4* haploinsufficiency could be explained by differences in the genetic background or in the levels of expression of the remaining allele. Further investigation and functional work incorporating additional patients with *ERBB4* variants is required to fully ascertain their consequence on protein function and resulting phenotype.

We propose that deletions that exclusively affect the intronic regions of *ERBB4* are less likely to be pathogenic. This is based on the following observations—individuals with the intronic deletions did not display consistent phenotypes, and intronic deletions were inherited from unaffected parents in several patients (Table 1) (Fig. 1). Furthermore, several other *ERBB4* intronic deletions are seen in control populations on the Database of Genomic Variants (DGV) and these observations are similar to what is observed with *NRXN1* exonic and intronic deletions [20, 21].

In summary, we demonstrate that 2q34 deletions that result in loss of exons of *ERBB4* may cause autosomal dominant mild to moderate developmental delay, ID or epilepsy. CNVs can help to identify novel disease-genes [22–24]. This study, therefore, adds *ERBB4* as a likely new candidate gene for ID. Future work will be required to identify if point variants in *ERBB4* also cause ID.
